# Age-Related Clinical Spectrum of *Plasmodium knowlesi* Malaria and Predictors of Severity

**DOI:** 10.1093/cid/ciy065

**Published:** 2018-03-05

**Authors:** Matthew J Grigg, Timothy William, Bridget E Barber, Giri S Rajahram, Jayaram Menon, Emma Schimann, Kim Piera, Christopher S Wilkes, Kaajal Patel, Arjun Chandna, Christopher J Drakeley, Tsin W Yeo, Nicholas M Anstey

**Affiliations:** 1 Global and Tropical Health Division, Menzies School of Health Research and Charles Darwin University, Darwin, Northern Territory, Australia; 2 Infectious Diseases Society Sabah–Menzies School of Health Research Clinical Research Unit, Malaysia; 3 Jesselton Medical Centre, Kota Kinabalu, Malaysia; 4 Clinical Research Centre, Queen Elizabeth Hospital, Kota Kinabalu, Malaysia; 5 Sabah Department of Health, Kota Kinabalu, Malaysia; 6 London School of Hygiene and Tropical Medicine, United Kingdom; 7 Lee Kong Chian School of Medicine, Nanyang Technological University, Singapore; 8 Communicable Disease Centre, Institute of Infectious Diseases and Epidemiology, Tan Tock Seng Hospital, Singapore

**Keywords:** *Plasmodium knowlesi*, malaria, district, clinical epidemiology, children

## Abstract

**Background:**

*Plasmodium knowlesi* is increasingly reported in Southeast Asia, but prospective studies of its clinical spectrum in children and comparison with autochthonous human-only *Plasmodium* species are lacking.

**Methods:**

Over 3.5 years, we prospectively assessed patients of any age with molecularly–confirmed *Plasmodium* monoinfection presenting to 3 district hospitals in Sabah, Malaysia.

**Results:**

Of 481 knowlesi, 172 vivax, and 96 falciparum malaria cases enrolled, 44 (9%), 71 (41%), and 31 (32%) children aged ≤12 years. Median parasitemia was lower in knowlesi malaria (2480/μL [interquartile range, 538–8481/μL]) than in falciparum (9600/μL; *P* < .001) and vivax malaria. In *P. knowlesi*, World Health Organization–defined anemia was present in 82% (95% confidence interval [CI], 67%–92%) of children vs 36% (95% CI, 31%–41%) of adults. Severe knowlesi malaria occurred in 6.4% (95% CI, 3.9%–8.3%) of adults but not in children; the commenst severity criterion was acute kideny injury. No patient had coma. Age, parasitemia, schizont proportion, abdominal pain, and dyspnea were independently associated with severe knowlesi malaria, with parasitemia >15000/μL the best predictor (adjusted odds ratio, 16.1; negative predictive value, 98.5%; *P* < .001). Two knowlesi-related adult deaths occurred (fatality rate: 4.2/1000 adults).

**Conclusions:**

Age distribution and parasitemia differed markedly in knowlesi malaria compared to human-only species, with both uncomplicated and severe disease occurring at low parasitemia. Severe knowlesi malaria occurred only in adults; however, anemia was more common in children despite lower parasitemia. Parasitemia independently predicted knowlesi disease severity: Intravenous artesunate is warranted initially for those with parasitemia >15000/μL.

## Teaser

Patients with knowlesi malaria were older and had lower parasitemia compared to human-only species. Severe knowlesi malaria occurred only in adults. Anemia was common in children despite lower parasitemia. Parasitemia predicted disease severity: Intravenous artesunate is warranted initially for parasitemia>15000/μL.

Since the initial description of a large focus of zoonotic *Plasmodium knowlesi* human cases in Sarawak, Malaysia, in 2004 [1], knowlesi malaria has been reported from countries across Southeast Asia [2, 3]. In Malaysia, *P. knowlesi* now accounts for >90% of all government-notified malaria cases [4–8], with >9500 reported cases from 2012 to 2016 [4, 5]. *Plasmodium knowlesi* is also increasingly reported in areas of western Indonesia [9, 10]. Difficulties with microscopic diagnosis [2, 11] have limited accurate reporting of the true incidence of knowlesi malaria, with the disease burden likely underestimated [2, 5–7, 12]. Despite great progress in reducing human-only malaria species in many countries [4, 5], increasing numbers of *P. knowlesi* cases in Southeast Asia threaten regional malaria elimination. Conventional public health measures are unable to target zoonotic transmission to humans from the *P. knowlesi* reservoir in monkey hosts, particularly outdoors in agricultural or forest areas [13–16].

Prospective studies have described the clinical spectrum of naturally acquired adult knowlesi malaria [17, 18]. Severe knowlesi malaria has been reported in adults in Southeast Asia and in adult travelers returning from these regions [2, 12, 18, 19], with the risk of severe disease at least as high as from *Plasmodium falciparum* [18]. Deaths from knowlesi malaria have been more common in older adults and have been associated primarily with respiratory distress, hypotension, and acute kidney injury (AKI) [6, 12, 19–21].

Malaria notification data in knowlesi-endemic areas show a median age of 31 years, much higher than that seen with *P. falciparum* and *Plasmodium vivax* [7], although 6% (79/1325) of all notified knowlesi malaria cases in Sabah in 2014 occurred in children aged <15 years [6]. With the marked reduction in cases of falciparum and vivax malaria, *P. knowlesi* now accounts for around 49% of all reported pediatric malaria cases in Sabah [6]. Despite this, there are limited descriptions of knowlesi malaria in children [2, 22], or comparisons between zoonotic knowlesi malaria and locally acquired malaria from the human-only species *P. falciparum* and *P. vivax* in district settings.

In this study, we compared the predefined clinical spectrum between children and adults with malaria due to *P. knowlesi* or other *Plasmodium* species infection, and evaluated predictors of disease severity in a coendemic primary care setting.

## METHODS

### Study Sites and Referral System

This study was conducted in Kudat Division, northwest Sabah, Malaysia, covering an area of 4623 km^2^ and with a total growth-rate adjusted Malaysian census–estimated population in 2016 of 199600 people. Each of the 3 districts in this division has a central referral hospital and subdistrict health clinics, consistent with other districts in Sabah. Malaysian Ministry of Health guidelines stipulate that all patients with fever receive microscopic blood slide screening for malaria parasites, with mandatory hospital admission, free treatment, and notification of positive cases [23].

### Subjects

Patients of all ages presenting to study hospitals with microscopy-diagnosed malaria were enrolled following written informed consent. Children were predefined as age ≤12 years, consistent with Malaysian Ministry of Health pediatric ward admission. Patients were not included in the final analysis if they were pregnant or had *Plasmodium malariae* infection on polymerase chain reaction (PCR), if *Plasmodium* species PCR was not confirmed, or if cross-check research microscopy was negative. A subset of patients with uncomplicated *P. knowlesi* and *P. vivax* malaria was also enrolled in previously reported randomized controlled treatment trials [23–25].

### Study Procedures

Baseline and longitudinal clinical, laboratory, and epidemiological data were entered using standardized case record forms. Venous blood was taken for baseline investigations and then daily for microscopy and hematology during hospital admission and at the follow-up visit 28 days after treatment initiation. Severe malaria was defined using World Health Organization (WHO) 2014 research criteria [26], including for *P. knowlesi*: hyperparasitemia threshold of 100000/μL, and jaundice defined as bilirubin >50 μmol/L with parasite count >20000/μL and/or creatinine >132 μmol/L [18]. Nonsevere anemia was defined using WHO age- and sex-based hemoglobin criteria [27]. AKI was evaluated using Kidney Disease Outcomes Quality Working Group (KDIGO) criteria [28]. Chronic disease was defined as hypertension; diabetes mellitus; ischemic heart disease; hyperlipidemia; or chronic kidney, liver, or respiratory disease.

### Laboratory Procedures

Microscopic asexual parasite and gametocyte counts were calculated by research microscopists using thick blood smears and quantitated leukocyte count. Standard hospital automated hematology, biochemistry, and microbiology laboratory results were used. Final *Plasmodium* species confirmation was done using PCR [29, 30].

### Statistical Analysis

We compared between-group differences with analysis of variance or Kruskal-Wallis testing for continuous variables, and Student *t* test or the Wilcoxon–Mann-Whitney test for 2-group comparisons according to distribution. For categorical variables, χ^2^ or Fisher’s exact test was used. Logistic regression models were fitted to determine a priori predictors of severe malaria based on standard clinical and laboratory WHO 2014 research criteria [26] evaluable at time of acute patient presentation to district hospital settings, including testing for model interactions and collinearity. Receiver operating characteristic (ROC) analysis was used to assess their sensitivity and specificity. Multivariate analysis controlled for age and log_e_ parasitemia; patients with hyperparasitemia as a sole severity criterion were considered nonsevere.

### Ethical Considerations

This study was approved by the medical research ethics committees of the Ministry of Health, Malaysia; London School of Hygiene and Tropical Medicine, United Kingdom; and Menzies School of Health Research, Australia.

## RESULTS

### Demographics

**Figure 1.**
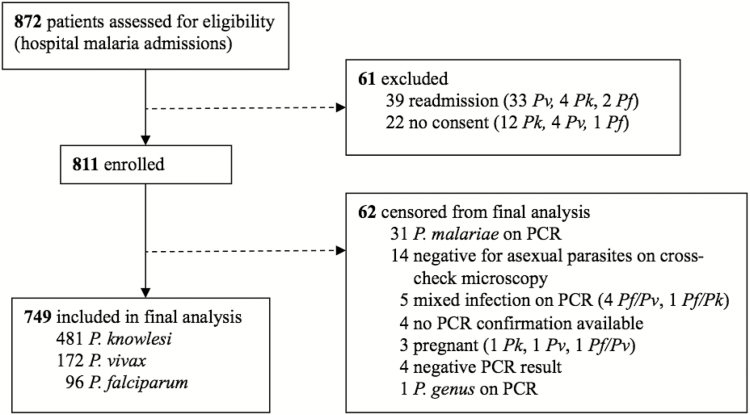
Enrollment flowchart. Abbreviations: PCR, polymerase chain reaction; *Pf*, *Plasmodium falciparum*; *Pk*, *Plasmodium knowlesi*; *Pv*, *Plasmodium vivax*.

**Figure 2.**
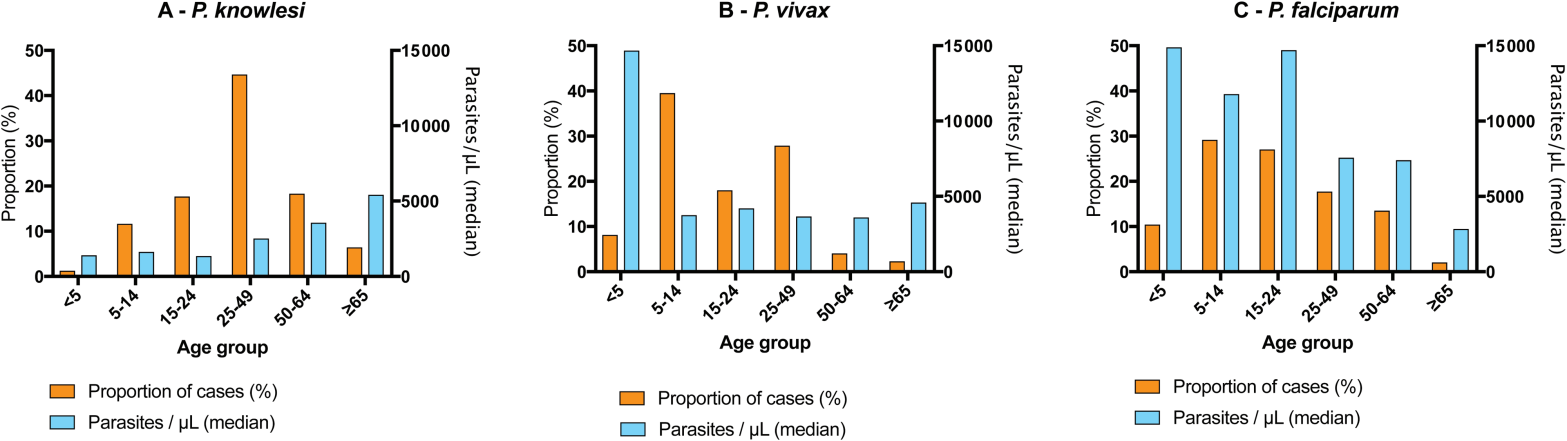
Proportion of cases and median parasite count by age-group and Plasmodium spp.

From October 2012 until April 2016, 811 malaria patients were enrolled (Figure 1). There were 481 *P. knowlesi*, 172 *P. vivax*, and 96 *P. falciparum* malaria cases included in the final analysis. From 2014 to 2015, the estimated minimum yearly malaria incidence in Kudat Division (district hospital presentations with clinical disease) for *P. knowlesi*, *P. vivax*, and *P. falciparum* was 0.79, 0.40, and 0.19 cases per 1000 people per year, respectively. Patients with knowlesi malaria had a median age of 33 years (interquartile range [IQR], 21–49 years), higher than those with vivax (15 years [IQR, 9–30 years]) and falciparum (16 years [IQR, 10–31 years]) malaria (*P* < .001; Figure 2). Patients aged >50 years comprised 107 (22%) of knowlesi malaria cases, compared to 10 (6%) and 14 (15%) for falciparum and vivax malaria, respectively (*P* < .001). A bimodal age distribution was seen for females with both *P. knowlesi* and *P. falciparum* infection. Of *P. knowlesi* cases, 44 (9%) were children, compared to 71 (41%) of *P. vivax* cases and 31 (32%) of those with *P. falciparum* malaria (Table 1; *P* < .001). Only 6 (1.3%) knowlesi cases were <5 years of age, with only 1 infant (<1 year), a 6-week-old with no travel history or forest or plantation exposure. Compared to children with *P. knowlesi* malaria, adults were more likely to be male (79% vs 57%; *P* = .001), with this relationship also evident for *P. vivax* cases (75% vs 48%; *P* < .001).

**Table 1.** Baseline Demographic, Clinical, and Laboratory Features of Children

Patient Characteristic	*Plasmodium knowlesi*	*Plasmodium vivax*	*Plasmodium falciparum*	*P* Value
Children (age ≤12 y), No. (% total)	44 (9.1)	71 (41.3)	31 (32.3)	<.001
Age, y				
Median (IQR)	8 (5–10)	9 (5–10)	7 (3–10)	.095
Range	0.1–12	0.67–12	1–12	
Male sex, No. (%)	25 (56.8)	34 (47.9)	21 (67.7)	.170
Previous malaria (self-reported), No. (%)	4 (9.1)	11 (15.5)	3 (9.7)	.526
History of chronic disease, No. (%)	2 (4.5)	0	0	.095
Days of fever	5 (3–7)	5 (3–7)	4 (3–5)	.751
Symptoms on enrollment, No. (%)				
Rigors	29 (65.9)	55 (77.5)	14 (45.2)	**.006**
Headache	34 (77.3)	55 (77.5)	21 (67.7)	.542
Vomiting	14 (31.8)	37 (52.1)	11 (35.5)	.068
Abdominal pain	19 (43.2)	9 (12.7)	9 (29.0)	**.001**
Diarrhea	4 (9.1)	2 (2.8)	4 (12.9)	.140
Cough	15 (34.1)	25 (35.2)	12 (38.7)	.914
Shortness of breath	3 (6.8)	4 (5.6)	4 (12.9)	.431
Myalgia	11 (25.0)	15 (21.1)	7 (22.6)	.890
Arthralgia	12 (27.3)	15 (21.1)	7 (22.6)	.746
Examination findings on enrollment				
Temperature, °C	37.1 (36.8–37.9)	37.4 (36.8–37.8)	37.1 (36.8–38)	.647
Fever (≥37.5°C), No. (%)	17 (38.6)	33 (46.5)	12 (38.7)	.634
Systolic blood pressure, mm Hg	101 (94–109)	102 (96–110)	106 (98–112)	.356
Heart rate, beats/min	104 (93–119)	105 (94–118)	117 (96–134)	.688
Respiratory rate, breaths/min	24 (22–27)	24 (22–28)	26 (24–28)	.081
Oxygen saturation, %	99 (99–100)	99 (98–100)	100 (99–100)	.504
Palpable liver, No. (%)	14 (31.8)	20 (28.2)	8 (25.8)	.842
Palpable spleen, No. (%)	9 (20.5)	12 (16.9)	2 (6.5)	.244
Rash, No. (%)	1 (2.3)	0 (0)	1 (3.2)	.360
Parasite count/μL	1722 (386–4830)	5967 (1829–13901)	7392 (1462–36546)	**<.001**
Parasite count/μL, range	36–74365	109–140500	61–635415	
Schizont proportion, mean % (SD)	3 (7.5)	1 (4.0)	0 (0)	**.013**
Schizont proportion >10%, No. (%)	3 (6.8)	1 (1.5)	0 (0)	.138
Parasite count >20000/μL, No. (%)	4 (9)	8 (11)	12 (39)	**.001**
Gametocytes present, no./No. (%)	4/35 (11)	21/66 (32)	1/11 (9)	**.035**
Hemoglobin, g/dL	10.6 (9.7–11.3)	10.1 (9.3–11.2)	10.3 (9.2–11.6)	.726
Anemia^a^ (baseline), No. (%)	36 (82)	56 (79)	21 (68)	.328
G6PD deficiency present, no./No. (%)	1/38 (2.6)	3/69 (4.3)	1/18 (5.6)	.852
White blood cell count, × 10^3^/μL	6.1 (5.1–7.5)	7.1 (5.1–8.6)	9.2 (6.4–12.7)	**.002**
Neutrophil count, × 10^3^/μL	2.7 (2.0–3.5)	3.3 (2.5–4.8)	3.8 (2.5–6.8)	**.015**
Lymphocyte count, × 10^3^/μL	2.0 (1.4–2.7)	2.2 (1.6–2.9)	2.8 (1.7–5.0)	**.025**
Monocyte count, × 10^3^/μL	1.1 (0.8–1.4)	0.9 (0.6–1.3)	1.2 (0.7–1.4)	.241
Platelet count, × 10^3^/μL	106 (80–163)	120 (93–179)	159 (83–282)	.078
Platelet nadir, × 10^3^/μL	78 (60–134)	104 (70–154)	129 (63–275)	**<.001**
Platelet nadir, d	1 (0–1)	1 (0–1)	1 (0–1)	1.000
Thrombocytopenia (platelets <150 × 10^3^/μL), No. (%)	30 (68)	46 (65)	13 (42)	**.047**
Creatinine, μmol/L	48 (36–57)	48 (34–58)	40 (31–53)	.267
Urea, mmol/L	3.8 (2.8–4.5)	3.5 (3.0–4.6)	3.2 (2.3–4.5)	.167
Sodium, mmol/L	137 (135–139)	137 (136–139)	136 (133–139)	.640
Bilirubin, μmol/L	11.5 (8.3–15.8)	9.8 (6.0–14.6)	11.0 (6.8–19.7)	.474
Glucose, mmol/L	5.8 (5.1–6.8)	5.7 (5.0–6.4)	6.2 (5.6–6.6)	.802
Albumin, g/dL	35 (31–37)	34 (28–36)	32 (29–37)	.644
AST, IU/L	25 (23–34)	24 (15–28)	37 (24–42)	.268
ALT, IU/L	16 (11–32)	17 (11–28)	24 (14–32)	.270
Bicarbonate, mmol/L	23 (21–25)	22 (20–26)	25 (21–26)	.418
Acute kidney injury, No. (%)	11 (26)	7 (10)	10 (32)	**.016**
Blood culture positive^b^, No. (%)	0/33 (0)	0/28 (0)	0/15 (0)	1.000

Data are presented as median (IQR) unless otherwise indicated. Results are from time of enrollment unless otherwise specified.

P values in bold font indicate a value <0.05.

Abbreviations: ALT, alanine aminotransferase; AST, aspartate aminotransferase; G6PD, glucose-6-phosphate dehydrogenase; IQR, interquartile range; SD, standard deviation.

^a^Anemia based on World Health Organization 2011 hemoglobin measurement criteria [27]: age 6–59 months (≤100 g/dL), 5–11 years (<115 g/dL), 12–14 years (<120 g/dL), nonpregnant women ≥15 years (<120 g/dL), pregnant women (<110 g/dL), men ≥15 years (<130 g/dL).

^b^Excluding results positive for skin contaminants.

### Baseline Features: Children

Abdominal pain was more common in children with knowlesi compared to vivax malaria (43% vs 13%; odds ratio [OR], 5.2 [95% confidence interval {CI}, 2.1–13.1]; *P* < .001), although vomiting occurred more often in those with *P. vivax* (*P* = .033) (Table 1). Children with knowlesi malaria had lower parasite counts than those with *P. vivax* (median, 1722 vs 5967 parasites/μL; *P* < .001) and *P. falciparum* (median, 1722 vs 7392 parasites/μL; *P* < .001). The highest parasite count recorded for a child with knowlesi malaria was 74365/μL, in an 11-year-old boy with uncomplicated disease. There were 36 (84%) children with knowlesi malaria with nonsevere anemia at presentation, comparable to children with other *Plasmodium* species infection, with no relationship to parasitemia demonstrated. The lowest hemoglobin level of 5.1 g/dL was seen in a 4-year-old child with knowlesi malaria 2 days after treatment, with 2 other children having minimum hemoglobin levels of 7.0 g/dL, all of whom had a parasite count <1000/μL at presentation. Children with knowlesi malaria had lower neutrophil and lymphocyte counts on presentation compared to those with other *Plasmodium* species (*P* = .002). Thirty (68%) children with knowlesi malaria had thrombocytopenia (platelet count <150 × 10^3^/μL), including 4 (9%) with a platelet count <50 × 10^3^ cells/μL. Frequency of thrombocytopenia in children with knowlesi malaria was comparable to those with *P. vivax* but more common than with *P. falciparum* malaria (OR, 3.0 [95% CI, 1.1–7.7]; *P* = .026). Children with knowlesi malaria were more likely to develop mild to moderate AKI compared to those with *P. vivax* (26% vs 10%; OR, 3.1 [95% CI, 1.1–8.7]; *P* = .030).

### Baseline Features: Adults

Duration of fever for *P. knowlesi*–infected adults was comparable to both children with *P. knowlesi* and adults with malaria due to other *Plasmodium* species (Table 2). Adult *P. knowlesi* cases were less likely to report abdominal pain compared to children with knowlesi malaria (23% vs 43%; OR, 0.40 [95% CI, .2–.8]; *P* = .004; Supplementary Table 1). Adults with knowlesi malaria had lower parasite counts (median, 2541/μL) than those with vivax (median, 3765/μL; *P* = .027) or falciparum (median, 9924/μL; *P* < .001) malaria (Table 2). Age was positively correlated with parasitemia in *P. knowlesi* (*r*^2^ = 0.15; *P* = .002), but not *P. falciparum* or *P. vivax* infection (Figure 2). Adult *P. knowlesi* cases had a lower risk of anemia at presentation compared to adults with vivax malaria (36% vs 50%, respectively; OR, 0.6 [95% CI, .4–.9]; *P* = .013), and children with knowlesi malaria (36% vs 82%; OR, 0.13 [95% CI, .06–.28]; *P* < .001). As with children, in adult knowlesi malaria parasitemia was not associated with anemia at enrollment after controlling for age. Adults with knowlesi malaria had lower platelet counts than those with other *Plasmodium* species (*P* < .001), with thrombocytopenia more common in adults compared to children with knowlesi malaria (92% vs 68%, respectively; *P* < .001). The risk of AKI was higher in adult *P. knowlesi* patients compared to *P. vivax* (19% vs 10%, respectively; OR, 2.1 [95% CI, 1.1–4.3]; *P* = .033), although this did not remain statistically significant after controlling for age; and was also comparable to that seen in both adults with *P. falciparum* and children with knowlesi malaria. Liver aminotransferases were higher in adults with knowlesi malaria compared to those with *P. vivax* including after controlling for age (*P* = .001). Of the 322 adults with knowlesi malaria who had blood cultures, only 1 grew a noncontaminant isolate, a 14-year-old with *Neisseria meningitidis*.

**Table 2.** Baseline Demographic, Clinical, and Laboratory Features in Adults

Patient Characteristic	*Plasmodium knowlesi*	*Plasmodium vivax*	*Plasmodium falciparum*	*P* Value
Adults (age >12 y), No. (% total)	437 (90.9)	101 (58.7)	65 (67.7)	**<.001**
Age, y				
Median (IQR)	35 (25–50)	27 (17–35)	24 (16–47)	**<.001**
Range	13–85	13–70	1–12	
Male sex, No. (%)	345 (78.9)	76 (75.2)	48 (73.8)	.522
Previous malaria (self-reported), No. (%)	93 (21.3)	26 (25.7)	8 (12.7)	.137
History of chronic disease, No. (%)	35 (8.0)	2 (2.0)	3 (4.6)	.066
Days of fever	4 (3–7)	5 (3–7)	4 (3–6)	.089
Symptoms on enrollment, No. (%)				
Rigors	359 (82.3)	86 (85.1)	49 (76.6)	.369
Headache	389 (89.0)	93 (92.1)	56 (86.2)	.469
Vomiting	105 (24.0)	43 (42.6)	28 (43.1)	**<.001**
Abdominal pain	102 (23.3)	25 (24.8)	18 (27.7)	.734
Diarrhea	36 (8.2)	10 (9.9)	7 (10.8)	.726
Cough	153 (35.0)	32 (31.7)	22 (33.8)	.814
Shortness of breath	70 (16.0)	20 (19.8)	10 (15.4)	.630
Myalgia	269 (61.6)	58 (57.4)	35 (53.8)	.418
Arthralgia	289 (66.1)	59 (58.4)	34 (52.3)	.052
Examination findings on enrollment				
Temperature, °C	37.4 (37.0–38.1)	37.4 (36.9–38.0)	37.0 (36.8–38)	**.001**
Fever (≥37.5°C), No. (%)	215 (49.3)	47 (46.5)	23 (35.4)	.634
Systolic blood pressure, mm Hg	120 (110–130)	115 (106–125)	112 (106–125)	**.004** ^**a**^
Heart rate, beats/min	88 (77–100)	92 (78–102)	92 (81–100)	**.025** ^**a**^
Respiratory rate, breaths/min	20 (20–24)	20 (20–22)	21 (20–22)	.260
Oxygen saturation, %	98 (97–99)	99 (98–100)	99 (98–100)	**<.001**
Palpable liver, No. (%)	105 (24.0)	21 (20.8)	11 (16.9)	.390
Palpable spleen, No. (%)	26 (5.9)	9 (8.9)	6 (9.2)	.404
Rash, No. (%)	19 (4.3)	3 (3.0)	1 (1.6)	.492
Parasite count/μL	2541 (478–8585)	3765 (1755–8122)	9924 (2522–22860)	**<.001**
Parasite count/μL, range	20–263772	53–184353	33–693922	
Schizont proportion, mean % (SD)	2 (5.4)	1 (2.1)	0 (0.1)	**<.001**
Schizont proportion >10%, No. (%)	32/432 (7.4)	2/99 (2.0)	0 (0)	**.014**
Parasite count >20000/μL, No. (%)	64 (15)	7 (7)	21 (32)	**<.001**
Gametocytes present, no./No. (%)	54/379 (14)	48/92 (52)	7/31 (23)	**<.001**
Hemoglobin, g/dL	13.2 (12.1–14.3)	12.8 (11.2–14.2)	13.1 (11.3–14.4)	.058
Anemia* (baseline), No. (%)	156 (36)	50 (50)	26 (41)	**.041**
G6PD deficiency present, no./No. (%)	4/364 (1.1)	4/94 (4.3)	1/48 (2.1)	.117
White blood cell count, × 10^3^/μL	6.1 (5.1–7.6)	6.5 (5.3–7.8)	6.5 (5.1–8.0)	**.013**
Neutrophil count, × 10^3^/μL	3.5 (2.6–4.5)	4.0 (3.0–5.1)	4.2 (3.1–5.4)	**<.001**
Lymphocyte count, × 10^3^/μL	1.4 (1.0–1.9)	1.4 (1.1–2.0)	1.4 (1.0–2.3)	.675
Monocyte count, × 10^3^/μL	1.0 (0.7–1.4)	0.7 (0.5–1.0)	0.7 (0.5–1.0)	**<.001**
Platelet count, × 10^3^/μL	70 (50–103)	95 (66–134)	91 (54–149)	**<.001**
Platelet nadir, × 10^3^/μL	60 (42–83)	85 (56–115)	81 (44–135)	**<.001**
Platelet nadir, d	1 (1-1)	1 (1-1)	1 (1-1)	1.000
Thrombocytopenia (platelets <150 × 10^3^/μL), No. (%)	401 (92)	82 (81)	49 (75)	**<.001**
Creatinine, μmol/L	88 (75–103)	78 (61–93)	75 (58–91)	**<.001**
Urea, mmol/L	5.2 (3.8–6.8)	4.7 (3.5–5.8)	5.0 (3.5–7.2)	**.032**
Sodium, mmol/L	136 (134–139)	137 (135–139)	136 (134–139)	.819
Bilirubin, μmol/L	17.1 (11.8–24.6)	14.0 (7.6–23.0)	18.3 (10.2–30.0)	**.032**
Glucose, mmol/L	6.4 (5.6–7.4)	6.3 (5.7–6.7)	6.3 (5.3–7.5)	.272
Albumin, g/dL	36 (30–40)	34 (30–38)	32 (29–37)	**.011**
AST, IU/L	34 (23–47)	10 (6–15)	28 (16–37)	**<.001**
ALT, IU/L	37 (24–56)	23 (14–36)	36 (22–48)	**<.001**
Bicarbonate, mmol/L	24 (21–27)	23 (21–25)	22 (20–24)	.921
Acute kidney injury, No. (%)	83 (19)	10 (10)	17 (27)	**.018**
Blood culture positive^b^, No. (%)	1/322 (<1)	0/64 (0)	0/31 (0)	1.000

Data are presented as median (IQR) unless otherwise indicated. Includes 2 *P. knowlesi* and 1 *P. falciparum* uncomplicated malaria adult patients given single-dose treatment by public health workers prior to enrollment.

P values in bold font indicate a value <0.05.

Abbreviations: ALT, alanine aminotransferase; AST, aspartate aminotransferase; G6PD, glucose-6-phosphate dehydrogenase; IQR, interquartile range; SD, standard deviation.

^*^Anemia based on World Health Organization 2011 hemoglobin measurement criteria [18]: age 6–59 months (≤100 g/dL), 5–11 years (<115 g/dL), 12–14 years (<120 g/dL), nonpregnant women ≥15 years (<120 g/dL), pregnant women (<110 g/dL), men ≥15 years (<130 g/dL).

^**a**^Comparisons did not remain statistically significant after controlling for age.

^b^Excluding results positive for skin contaminants; 1 patient with knowlesi malaria was positive for *Neisseria meningitidis.*

### Severe Malaria

*Plasmodium knowlesi* was the most common cause of severe malaria, with 28 of 481 (5.8%) knowlesi cases having severe disease (Table 3), all of whom were adults (28/437; 6.4% [95% CI, 3.9%–8.3%]. Of the severe knowlesi cases, 19 (68%) had severe malaria on presentation and 9 (32%) developed severe complications following commencement of treatment. A single severity criterion defined severe malaria in 16 (57%), with 12 (43%) patients having ≥2 criteria (Table 4). The most common severity criterion was severe AKI (creatinine >265 μmol/L), occurring in 10 (35.7%) severe knowlesi patients, including a single patient who progressed to severe AKI on day 1 of admission. Eight (29%) knowlesi patients had hyperparasitemia >100000/μL, including 5 (18%) as a sole severity criterion. Parasite counts were higher in severe knowlesi compared to nonsevere knowlesi malaria (median, 42224 vs 2044 parasites/μL, respectively; *P* < .001). The platelet count was lower in severe vs uncomplicated knowlesi malaria (median, 56 vs 75 × 10^3^ cells/μL, respectively; *P* = .004), neutrophil count was higher (median, 4.9 vs 3.7 × 10^3^ cells/μL, respectively; *P* = .004), and proportion of patients with hyponatremia was higher (48% vs 28%, respectively; *P* = .028). Five (18%) knowlesi patients had documented hypotension, all of whom had other severity criteria including 2 patients with respiratory distress. Empiric antibiotic treatment was given to 9 (32%) patients with severe knowlesi malaria. Of the 4 severe *P. vivax* cases, 2 were children with hyperbilirubinemia and a parasite count >20000/μL, both with moderate anemia (hemoglobin nadir of 6.8 g/dL and 9.1 g/dL, respectively). The other 2 patients with severe vivax malaria were adults, including a 17-year-old female with severe anemia, and a 53-year-old man with hypotension and respiratory distress. There were 5 patients with severe falciparum malaria (5.2%), including 2 children. No patient with malaria from any *Plasmodium* species had coma.

**Table 3.** Severe Malaria

Characteristic	Plasmodium knowlesi (N = 481)	*Plasmodium vivax*(N = 172)	*Plasmodium falciparum*(N = 96)	*P* Value
Severe cases				
No.	28	4	5	
%	5.8	2.3	5.2	.225
95% CI	3.9–8.3	0.6–5.8	1.7–11.7	
Age, y				
Median	53	11	14	**<.001**
IQR	43–64	4–35	2–16	
Range	13–78	3–53	1–26	
Child age ≤12 y				
No.	0	2	2	**.001**
%	0	2.8	6.5	
Male sex				
No.	19	2	4	.709
% severe	68	50	80	
Parasitemia/μL				
Median	42225	19333	297000	**.031**
IQR	17221–103577	6076–43680	85505–635415	

P values in bold font indicate a value <0.05.

Abbreviations: CI, confidence interval; IQR, interquartile range.

**Table 4.** Severe *Plasmodium knowlesi* Malaria Characteristics

WHO Severity Criteria	Definition	No.	% Severe (n = 28)	% Total (n = 481)
Hyperparasitemia	Parasite count >100000/μL	8	28.6	1.7
Hypotension	Systolic blood pressure <70 mm Hg in children or <80 mm Hg in adults	5	17.9	1.0
Impaired consciousness	Glasgow coma score <11 in adults or Blantyre coma score <3 in children	0	0	0
Metabolic acidosis	Plasma bicarbonate <15 mmol/L	3	10.7	0.6
Respiratory distress	Oxygen saturation <92% on room air with a respiratory rate >30/min	2	7.1	0.4
Jaundice	Total bilirubin >50 μmol/L; with parasite count >20000/μL and/or creatinine level >132 μmol/L	8	28.6	2.0
Severe acute kidney injury	Plasma or serum creatinine >265 μmol/L	10	35.7	2.1
Severe malarial anemia^a^	Hemoglobin concentration <5 g/dL in children, and <7 g/dL in adults	8	28.6	1.7
Hypoglycemia	Blood or plasma glucose <2.2 mmol/L	1	3.6	0.2
Significant bleeding	Including recurrent or prolonged bleeding from nose, gums, or venipuncture sites; hematemesis or melena	1	3.6	0.2
≥2 criteria		12	42.9	2.5
Severe criteria developed after presentation		8	28.6	1.7
Transfer to tertiary hospital		15	53.6	3.1
Admitted to ICU		10	35.7	2.1
Death		2	7.1	0.4

P values in bold font indicate a value <0.05.

Abbreviations: ICU, intensive care unit; WHO, World Health Organization.

^a^Includes 2 *Plasmodium knowlesi* patients with serious underlying medical illness: 1 with worsening of known chronic kidney disease (acute kidney injury) and another with endometriosis-associated bleeding (anemia).

### Predictors of Severe Malaria

On multivariate logistic regression controlling for age and parasitemia, independent clinical and parasitological predictors of severe disease in knowlesi malaria included schizont proportion >10%, abdominal pain, and dyspnea (Table 5). Among WHO laboratory severity criteria, creatinine, hemoglobin, bicarbonate, and bilirubin remained independent predictors of severe disease when patients with only a single WHO severity criterion based on these measures were reclassified as nonsevere. A parasite threshold of 15000/μL had the best-combined sensitivity (74%) and specificity (87%) for predicting severe knowlesi malaria, with an area under the curve of 0.80 (95% CI, .71–.90) and a negative predictive value of 98.5%. Age ≥45 years was the best predictor of hyperparasitemia when controlling for other variables (adjusted OR, 4.9 [95% CI, 1.0–23.9]; *P* = .048). Adults with knowlesi malaria had a higher risk of severe disease compared to adult patients with vivax malaria (OR, 3.4 [95% CI, .8–14.5]; *P* = .098), and a comparable risk to falciparum malaria.

**Table 5.** Selected Clinical and Laboratory Predictors of Severe Knowlesi Malaria at Presentation

Variable	UM	SM	Univariate Analysis^a^			ROC Analysis						Multivariate Analysis^b^		
	(n = 453)	(n = 28)												
	%	%	OR	(95% CI)	*P* Value	Sens (%)	Spec (%)	PPV (%)	NPV (%)	AUC	(95% CI)	aOR	(95% CI)	*P* Value
Age >40 y	37	79	6.3	(2.5–16.0)	<.001	79	63	11.7	98.0	0.71	(.63–.79)	5.7	(1.8–17.6)	**.003**
Age >45 y	28	71	6.5	(2.8–15.1)	<.001	71	72	13.7	97.6	0.72	(.63–.81)	7.3	(2.6–21.0)	**<.001**
Age >50 y	20	57	5.3	(2.4–11.6)	<.001	57	80	15.0	96.8	0.69	(.59–.78)	4.5	(1.8–11.4)	**.001**
Male sex	23	68	1.6	(.7–3.7)	.245	32	78	8.1	94.9	0.55	(.46–.64)	1.7	(.6–5.0)	.312
Previous malaria episode	19	36	2.3	(1.0–5.2)	.039	36	81	10.3	95.3	0.57	(.47–.66)	2.4	(.9–6.6)	.530
Chronic disease	6	29	5.9	(2.4–14.4)	<.001	29	94	21.6	95.5	0.61	(.52–.70)	2.3	(.7–7.4)	.170
Abdominal pain	24	46	2.8	(1.3–6.0)	.010	46	76	10.7	95.8	0.61	(.52–.71)	3.5	(1.3–9.2)	**.013**
Shortness of breath	14	39	4.1	(1.8–9.1)	.001	39	86	15.1	95.8	0.63	(.54–.72)	4.1	(1.5–11.0)	**.005**
Parasite count >10000/μL	17	79	12.6	(4.8–33.0)	<.001	74	82	21.8	98.4	0.78	(.68–.87)	10.0	(3.7–26.8)	**<.001**
Parasite count >15000/μL	12	79	18.8	(7.1–49.6)	<.001	74	87	28.6	98.5	0.80	(.71–.90)	16.1	(5.9–44.0)	**<.001**
Parasite count >20000/μL	11	64	11.6	(4.8–28.2)	<.001	61	88	26.5	97.6	0.75	(.64–.85)	8.9	(3.5–27.6)	**<.001**
Parasite count >35000/μL	5	57	18.1	(7.3–45.0)	<.001	52	94	42.1	97.3	0.73	(.63–.84)	12.8	(4.9–33.6)	**<.001**
Schizont proportion >1%	31	57	2.9	(1.4–6.4)	.006	57	69	57.1	68.8	0.63	(.53–.73)	1.4	(.5–3.6)	.510
Schizont proportion >10%	8	18	2.7	(1.0–7.5)	.060	18	92	17.9	92.5	0.55	(.48–.63)	5.3	(1.5–19.4)	**.011**
Platelets <30 × 10^3^/μL	6	18	3.7	(1.3–10.5)	.014	18	94	17.9	94.4	0.56	(.49–.63)	1.4	(.4–5.0)	.623
Platelets <50 × 10^3^/μL	22	43	2.6	(1.2–5.7)	.015	43	78	10.7	95.6	0.60	(.51–.70)	1.2	(.5–3.1)	.703
Bilirubin >25 μmol/L	21	50	3.3	(1.2–8.7)	.018	47	79	9.1	97.0	0.63	(.50–.75)	1.9	(.6–5.6)	.244
Bilirubin >30 μmol/L	14	46	4.1	(1.5–11.3)	.006	41	86	11.3	97.0	0.63	(.51–.76)	2.3	(.7–7.0)	.147
Bilirubin >50 μmol/L	3	33	10.9	(3.4–35.2)	<.001	29	96	26.3	96.8	0.63	(.52–.74)	5.1	(1.4–19.4)	**.016** ^c^
Creatinine >100 μmol/L	24	61	4.3	(1.9–9.6)	<.001	58	76	12.3	69.8	0.67	(.57–.77)	3.1	(1.1–8.6)	**.028** ^c^
Creatinine >132 μmol/L	5	28	14.9	(6.2–35.7)	<.001	46	95	33.3	96.8	0.70	(.61–.80)	6.6	(2.3–19.2)	**.001** ^c^
Creatinine >150 μmol/L	3	43	17.8	(6.9–45.6)	<.001	38	97	40.0	96.4	0.68	(.58–.77)	6.9	(2.1–22.1)	**.001** ^c^
Hemoglobin <9 g/dL	2	29	7.4	(2.2–24.9)	.001	17	97	25.0	95.7	0.57	(.49–.65)	13.5	(2.4–75.6)	**.003** ^c^
Hemoglobin <10 g/dL	6	36	4.6	(1.7–12.3)	.003	25	93	16.2	95.9	0.59	(.50–.68)	8.1	(2.2–29.8)	**.002** ^c^
Anemia (WHO age criteria)	34	64	4.1	(1.5–11.0)	.005	68	65	7.6	98.0	0.67	(.56–.78)	4.1	(1.4–12.5)	**.012** ^c^
Neutrophil count >4.0 × 10^3^/μL	34	60	2.9	(1.3–6.5)	.013	60	66	9.0	96.6	0.63	(.53–.73)	1.3	(.5–3.4)	.661
Bicarbonate <20 mmol/L	15	38	3.4	(1.4–8.6)	.009	38	85	12.7	95.9	0.61	(.51–.72)	2.4	(.8–7.3)	.128
Bicarbonate <18 mmol/L	1	24	22.2	(5.8–84.5)	<.001	24	99	50.0	95.7	0.61	(.52–.71)	19.6	(2.9–132)	**.002** ^c^
Age ≥45 and parasite count >15000/μL	5	68	43.3	(16.4–114.6)	<.001	65	96	44.1	98.2	0.81	(.71–.91)	…		
Age ≥45 or parasite count >15000/μL	46	89	10.6	(3.1–36.1)	<.001	87	61	10.2	98.9	0.74	(.67–.82)	…		
Age ≥50 or parasite count >20000/μL	3	50	8.4	(3.1–21.2)	<.001	78	70	11.6	98.5	0.74	(.65–.83)	…		
Age ≥45 or chronic disease	31	79	8.0	(3.2–20.2)	<.001	79	69	13.4	98.1	0.74	(.66–.82)	6.6	(2.4–17.8)	**<.001**

All odds ratios are presented using the complement within the defined variable, for example, age >40 years compared with ≤40 years.

P values in bold font indicate a value <0.05.

Abbreviations: aOR, adjusted odds ratio; AUC, area under the curve; CI, confidence interval; NPV, negative predictive value; OR, odds ratio; PPV, positive predictive value; ROC, receiver operating characteristic; Sens, sensitivity; SM, severe malaria; Spec, specificity; UM, uncomplicated malaria; WHO, World Health Organization.

^a^Univariate analysis: patients with only a single severity criterion (hyperparasitemia, acute kidney injury, anemia, respiratory distress, acidosis, or jaundice) were considered nonsevere for the relevant analysis of the corresponding clinical or investigation result.

^b^Multivariate analysis controlled for: age and ln(parasitemia). Patients with hyperparasitemia as a single severity criterion were considered nonsevere.

^c^Creatinine, hemoglobin, bicarbonate, and bilirubin at the thresholds shown remained independent predictors of severe disease when patients with only a single WHO severity criterion based on these markers were reclassified as nonsevere.

### Case Fatalities

There were 2 deaths attributed to malaria, both *P. knowlesi*, giving an overall *P. knowlesi* case fatality risk of 2 of 481 (0.4% [95% CI, 0.1%–1.5%]), or 2 of 437 (0.5% [95% CI, 0.1%–1.6%]) in adults. The *P. knowlesi*–related deaths were a 62-year-old woman with hyperparasitemia (263772/μL) and moderate AKI (creatinine: 224 μmol/L), who developed hypotension and acute respiratory distress [6], and a 50-year-old man presenting with severe AKI (creatinine: 609 μmol/L), parasitemia of 71939/μL, and moderate anemia (hemoglobin: 9.9 g/dL).

## DISCUSSION

This study is the largest series of *P. knowlesi* malaria cases to date, and the first to prospectively compare the clinical spectrum of disease between adults and children. Although 91% of knowlesi malaria cases were adults, morbidity in children was also demonstrated, with an 11-fold higher risk of anemia at presentation and a similar risk of mild to moderate AKI compared to adults [1, 7]. The majority of adults with knowlesi malaria had uncomplicated disease and, compared to those with vivax and falciparum malaria, were older, with a lower risk of nonsevere anemia and a higher risk of thrombocytopenia, consistent with previous reports [1, 17, 18]. The lower parasitemia in both children and adults with clinical illness from *P. knowlesi* infection compared with the human-only *Plasmodium* species may indicate a lower pyrogenic threshold and greater inflammatory response, consistent with poor adaptation of this zoonotic parasite to the human host. Although *P. knowlesi* has a 24-hour blood-stage life cycle in humans, the low parasitemia in most infections may indicate variable efficiency in human red blood cell (RBC) invasion [31]. Only a minority had high parasitemia, with parasitemia an independent predictor of severe knowlesi malaria overall. Notably, there was no coma or convulsions seen in any patient with knowlesi malaria, consistent with previous studies [6, 17, 18, 32]. No child with knowlesi malaria had severe manifestations (although borderline severe anemia was present in one), in contrast to the severe disease found in pediatric falciparum and vivax malaria in this series and elsewhere [26].

A lower proportion of *P. knowlesi* infections were in children compared to those with *P. vivax* or *P. falciparum*. The lower incidence of clinical disease from *P. knowlesi* infection in infants and also older children has been attributed to epidemiological factors such as lower forest exposure [1, 13, 18], although contributing age-related innate protective mechanisms are plausible [33], and asymptomatic infection has been reported in children [34]. Most children with knowlesi malaria had anemia at enrollment, consistent with a previous retrospective report [22]. Although adults with knowlesi malaria had higher parasite counts, nonsevere anemia was more common in children, suggesting that children may have a higher rate of uninfected RBC destruction and/or greater dyserythropoiesis, although underlying mechanisms and baseline community anemia prevalence require further investigation [35]. Children with knowlesi malaria had lower parasitemia and platelet counts compared to children with either *P. vivax* or *P. falciparum* infection, in addition to a lower neutrophil count compared to *P. vivax*. However, there was a comparable risk of nonsevere anemia and AKI seen in *P. knowlesi*–infected children as in those with *P. vivax* or *P. falciparum*.

The proportion of adults with severe disease from *P. knowlesi* infection was comparable to that seen in *P. falciparum*. The risk of severe knowlesi malaria in this primary referral setting in Sabah, 6.2% in adults, was similar to district hospital presentations in Sarawak (9.3%) [17], and lower than that demonstrated in a tertiary hospital setting of 29% [18]. Severe AKI was the most frequent severity criterion, and has commonly been reported in other adult studies [17, 18, 32]. Severe anemia was present in a larger proportion of adults with severe knowlesi malaria than in a previous tertiary-referral study, which reported anemia as a severe criterion in only 5% of adults, 1 of whom was splenectomized [18]. *Plasmodium knowlesi* parasitemia [18, 32] and age [33] independently predicted severe disease in this study, in addition to abdominal pain and dyspnea, which have not been previously demonstrated. Parasite counts were higher in severe knowlesi malaria than in uncomplicated disease despite no difference in the number of preceding days of fever, which suggests differences in efficacy and tropism of normocyte invasion and parasite multiplication [31]. With age an independent risk factor for both parasitemia and severity, the immunosenescence that occurs with aging [33, 36] may also result in impaired control of parasite multiplication.

The pathophysiological mechanisms in severe knowlesi malaria are not well understood but likely differ from *P. falciparum*, with coma remaining unreported and a lack of the retinal microcirculatory changes found in severe falciparum malaria [37]. With endothelial activation and systemic inflammation at least as high in response to *P. knowlesi* as in *P. falciparum* infection [33, 36], these processes also likely contribute to pathogenesis, particularly with the comparatively low parasite biomass able to produce severe disease observed in this study. The nature and role of microvascular accumulation of parasitized RBCs, a key mechanism of severe knowlesi malaria in rhesus macaques and also observed in a single human autopsy report [21], requires investigation. RBC deformability is reduced in proportion to disease severity in knowlesi malaria [38], however the role of hemolysis and endothelial dysfunction, other key pathogenic mechanisms also present in severe falciparum malaria [39], require further investigation. Phenotypic glucose-6-phosphate dehydrogenase deficiency has been shown to protect against knowlesi malaria [13]. Other host genetic factors related to selection pressure from historical human-only *Plasmodium* transmission may also modulate disease severity.

Current knowlesi malaria management guidelines in Sabah recommend referral for tertiary care and initial treatment with intravenous artesunate for any patients >50 years of age or with a parasitemia >20000/μL [18, 40]. Along with appropriate intravenous artesunate administration for severe malaria due to any *Plasmodium* species, these management guidelines have contributed to a decline in reported malaria case-fatality rate [6, 18, 20]. In the current study, predictors were limited to severe disease given the low case-fatality rate, with a parasite threshold of 15000 parasites/μL giving a high negative predictive value of 98.5%. A conservative approach would be to recommend early administration of intravenous artesunate initially for any knowlesi malaria case with a parasite count above this threshold, given the potential delay or inability to evaluate other laboratory markers of severe disease in most primary care settings.

In conclusion, although the majority of cases are uncomplicated, *P. knowlesi* infection causes morbidity at comparatively low parasitemia in both adults and children. Adults are at risk of severe and fatal disease, in contrast to children, among whom this was not demonstrated. A conservative treatment approach utilizing parasite counts to predict severe disease is warranted.

## Supplementary Data

Supplementary materials are available at *Clinical Infectious Diseases* online. Consisting of data provided by the authors to benefit the reader, the posted materials are not copyedited and are the sole responsibility of the authors, so questions or comments should be addressed to the corresponding author.

Supplementary Table 1

## Notes

***Author contributions.*** N. M. A., T. W. Y., T. W., B. E. B., and M. J. G. conceived and designed the study; M. J. G., E. S., C. S. W., K. P., and A. C. conducted the study with assistance from G. S. R., T. W. Y., T. W., J. M., B. E. B., C. J. D., and N. M. A; K. P. coordinated the laboratory work; M. J. G. conducted the data analysis, with assistance from N. M. A., T. W. Y., and B. E. B; M. J. G. wrote the first draft of the manuscript. All authors reviewed the final manuscript.

***Acknowledgments.*** We thank the study participants; the malaria research team nursing and laboratory staff; and Malaysian Ministry of Health hospital directors and clinical staff at Kudat, Kota Marudu, and Pitas District Hospitals. We also recognize the support from Dr Goh Pik Pin and the Clinical Research Centre, Queen Elizabeth Hospital, Kota Kinabalu, Sabah; colleagues and staff of the UK Medical Research Council *P. knowlesi* Monkeybar Project; and the Director-General of Health, Ministry of Health, Malaysian government. We thank Amar Aziz (Menzies School of Health Research) for conducting the polymerase chain reaction assays.

***Financial support*** This study was funded by the Malaysian Ministry of Health (grant number BP00500420); Asia Pacific Malaria Elimination Network (grant number 108-07); Australian National Health and Medical Research Council (grant numbers 1037304 and 1045156; fellowships [numbers 1042072 to N. M. A., 605831 to T W.Y., 1088738 to B. E. B., and 1138860 to M. J. G.]; “Improving Health Outcomes in the Tropical North: a multidisciplinary collaboration ‘Hot North” (grant number 1131932; fellowship to M. J. G.); the UK Medical Research Council, Natural Environment Research Council, Economic and Social Research Council, and Biotechnology and Biosciences Research Council (Environmental and Social Ecology of Human Infectious Diseases Initiative; grant number G1100796); and the National Institutes of Health (USA) grant number R01AI116472-01. T. W. Y. is funded by a Singapore National Medical Research Council Clinician Scientist Award (CSA INV 15nov007).

***Potential conflicts of interest.*** All authors: No reported conflicts of interest. All authors have submitted the ICMJE Form for Disclosure of Potential Conflicts of Interest. Conflicts that the editors consider relevant to the content of the manuscript have been disclosed.
