# Supplementary material for: Age-Related Clinical Spectrum of Plasmodium knowlesi Malaria and Predictors of Severity
Source: Clin Infect Dis. 2018 Mar 5;67(3):350–9. doi: 10.1093/cid/ciy065 (PMC6051457; doi:10.1093/cid/ciy065)
Supplement: Supplementary Table 1 [file ciy065_suppl_supplementary_table_1.docx]

**Supplementary Table 1.**

Baseline clinical and laboratory features – *P. knowlesi* cases: children (≤12 years) versus adults

| **Patient characteristic** | ***Children***  n=44 (9.1) | ***Adults***  n=437 (90.9) | **P-value** |
| --- | --- | --- | --- |
|  |  |  |  |
| Age, years |  |  |  |
| Median (IQR) | 8 (5-10) | 35 (25-50) | - |
| Range | 0.1-12 | 13-85 |  |
|  |  |  |  |
| Male gender, n (%) | 25 (56.8) | 345 (78.9) | **0.001** |
|  |  |  |  |
| Previous malaria (self-reported), n (%) | 4 (9.1) | 93 (21.3) | 0.055 |
|  |  |  |  |
| History of chronic disease, n (%) | 2 (4.5) | 35 (8.0) | 0.411 |
|  |  |  |  |
| Days of fever, median (IQR) | 5 (3-7) | 4 (3-7) | 0.496 |
|  |  |  |  |
| Symptoms on enrolment, n (%) |  |  |  |
| Rigors | 29 (65.9) | 359 (82.3) | **0.008** |
| Headache | 34 (77.3) | 389 (89.0) | **0.023** |
| Vomiting | 14 (31.8) | 105 (24.0) | 0.254 |
| Abdominal pain | 19 (43.2) | 102 (23.3) | **0.004** |
| Diarrhoea | 4 (9.1) | 36 (8.2) | 0.776 |
| Cough | 15 (34.1) | 153 (35.0) | 0.903 |
| Shortness of breath | 3 (6.8) | 70 (16.0) | 0.105 |
| Myalgia | 11 (25.0) | 269 (61.6) | **<0.001** |
| Arthralgia | 12 (27.3) | 289 (66.1) | **<0.001** |
|  |  |  |  |
| Examination findings on enrolment |  |  |  |
| Temperature, °C, median (IQR) | 37.1 (36.8-37.9) | 37.4 (37.0-38.1) | 0.105 |
| Fever, temp≥37.5°C | 17 (38.6) | 215 (49.3) | 0.181 |
| Systolic blood pressure, mmHg, mean (SD) | 103 (12.7) | 120 (16.2) | **-** |
| Heart rate, beats/min, mean (SD) | 105 (18.3) | 89 (16.9) | **-** |
| Respiratory rate, breaths/min, mean (SD) | 25 (4.4) | 21 (2.4) | **-** |
| Oxygen saturation, %, median (IQR) | 99 (99-100) | 98 (97-99) | **-** |
| Palpable liver, n (%) | 14 (31.8) | 105 (24.0) | - |
| Palpable spleen, n (%) | 9 (20.5) | 26 (5.9) | **-** |
| Rash, n (%) | 1 (2.3) | 19 (4.3) | 0.511 |
|  |  |  |  |
| Parasite count, parasites/μL, median (IQR) | 1722 (386-4830) | 2541 (478-8585) | 0.176 |
| Parasite count, parasites/μL, range | 36-74365 | 20-263772 |  |
| Parasite count, geometric mean (95%CI) | 1511 (1671-9158) | 2185 (8132-12762) | 0.176 |
| Trophozoite proportion, mean % (SD) | 97 (7.4) | 98 (5.4) | 0.642 |
| Schizont proportion, mean % (SD) | 3 (7.5) | 2 (5.4) | 0.643 |
| Schizont proportion >10%, n (%) | 3 (6.8) | 32/432 (7.4) | 0.742 |
| Parasite count >20,000/μL, n (%) | 4 (9) | 64 (15) | 0.373 |
| Gametocytes present, n/N (%) | 4/35 (11) | 54/379 (14) | 0.646 |
|  |  |  |  |
| Haemoglobin, g/dL, mean (SD) | 10.4 (1.4) | 13.1 (1.8) | 0.726 |
| Anaemia (baseline), n (%) | 36 (82) | 156 (36) | **<0.001** |
|  |  |  |  |
| G6PD deficiency present, n/N (%) | 1/38 (2.6) | 4/364 (1.1) | 0.393 |
|  |  |  |  |
| White blood cell count, ×10^3^/μL, median (IQR) | 6.1 (5.1-7.5) | 6.1 (5.1-7.6) | 0.972 |
| Neutrophil count, ×10^3^/μL, median (IQR) | 2.7 (2.0-3.5) | 3.5 (2.6-4.5) | **<0.001** |
| Lymphocyte count, ×10^3^/μL, median (IQR) | 2.0 (1.4-2.7) | 1.4 (1.0-1.9) | **<0.001** |
| Monocyte count, ×10^3^/μL, median (IQR) | 1.1 (0.8-1.4) | 1.0 (0.7-1.4) | 0.500 |
|  |  |  |  |
| Platelet count, ×10^3^/μL, median (IQR) | 106 (80-163) | 70 (50-103) | **<0.001** |
| Platelet nadir, ×10^3^/μL, median (IQR) | 78 (60-134) | 60 (42-83) | **<0.001** |
| Platelet nadir, median days (IQR) | 1 (0-1) | 1 (1-1) | 0.968 |
| Thrombocytopenia (platelets <150×10^3^/μL) | 30 (68) | 401 (92) | **<0.001** |
|  |  |  |  |
| Creatinine, μmol/L, median (IQR) | 48 (36-57) | 88 (75-103) | **-** |
| Urea, mmol/L, median (IQR) | 3.8 (2.8-4.5) | 5.2 (3.8-6.8) | **<0.001** |
| Sodium, mmol/L, median (IQR) | 137 (135-139) | 136 (134-139) | 0.659 |
| Bilirubin, μmol/L, median (IQR) | 11.5 (8.3-15.8) | 17.1 (11.8-24.6) | **<0.001** |
| Glucose, mmol/L, median (IQR) | 5.8 (5.1-6.8) | 6.4 (5.6-7.4) | **0.003** |
| Albumin, g/dL, median (IQR) | 35 (31-37) | 36 (30-40) | 0.292 |
| AST, IU/L, median (IQR) | 25 (23-34) | 34 (23-47) | 0.323 |
| ALT, IU/L, median (IQR) | 16 (11-32) | 37 (24-56) | **<0.001** |
| Bicarbonate, mmol/L, median (IQR) | 21 (20-23) | 24 (21-27) | **0.001** |
|  |  |  |  |
| Acute kidney injury, n (%) | 11 (26) | 83 (19) | 0.215 |
|  |  |  |  |
| Blood cultures positive*, n (%) | 1/33 (3%) | 0/322 (0%) | **0.002** |
|  |  |  |  |

Results are from time of enrolment unless otherwise specified

Includes 2 *P. knowlesi* uncomplicated adult malaria patients given single dose treatment by public health workers prior to presentation at hospital

*Excluding results positive for skin contaminants
